# Adult Hospitalists’ Knowledge of Radiation Oncology in an Independent Nonacademic Healthcare System in Albuquerque, New Mexico

**DOI:** 10.7759/cureus.41856

**Published:** 2023-07-13

**Authors:** Elvina C Lingas, Venu M Ganipisetti

**Affiliations:** 1 Hospital Medicine, Presbyterian Hospital, Albuquerque, USA

**Keywords:** quality improvement research, hospitalist medicine, cancer education, radiation & medical oncology, radiation oncology education

## Abstract

Radiation oncology (RO) plays a crucial role in cancer care; cancer patients often undergo their initial diagnostic study by general physicians. However, studies have shown that most physicians are uncomfortable referring cancer patients to radiation therapy (RT). To assess the knowledge of RO among non-oncology physicians, we sent invitations to complete an online survey that required the responders to self-rate their understanding of RT and objective assessment. The survey was targeted at hospitalists and primary care physicians. Forty physicians responded to the survey, and 89.7% practiced primarily as hospitalists, with 67% being Internal Medicine graduates. Fifty percent of physicians have referred patients to RO before, although more than 90% have not done additional CME (continuing medical education) in Oncology. More than 50% of recent graduates (one to five years post-residency) self-rated themselves as “not knowledgeable” when it comes referral process to RO as well as general knowledge regarding RT. Factors, such as “type of cancer,” “patients' wishes,” and “life expectancy,” are most cited as factors influencing the decision for a referral.

## Introduction

Radiation oncology (RO) continues to be an essential aspect of cancer care [1], with half of the patients receiving radiation therapy (RT) in the course of their illness as well as contributing to 40% of curative treatment [2]. Aside from curative intent [3-5], RT is also effective for palliative purposes, such as decreasing pain from bone metastasis [6].

Despite the established benefits of RO and significant advances in recent years, there is a distinctive lag in RO education and familiarity among residents [7], even among attendings who care for cancer patients [8,9]. This could create a delay in referrals due to insufficient understanding. Several studies have proposed that introducing the RO module to medical students increased their knowledge of the topic and interest in pursuing further training [10-12].

Approximately four million cancer patients visit the emergency department (ED) every year in the United States only for cancer-related complications [13], and most of these patients end up being hospitalized [14]. Hospitalists' role in managing inpatient care of cancer patients is well established. Despite this, there are only a few studies assessing knowledge about RO among hospitalists.

In this study, the authors surveyed the knowledge of RO among hospitalists and primary care physicians in a large, independent, nonacademic healthcare system in Albuquerque, New Mexico, and the factors influencing their referral.

## Materials and methods

Research tool

We used Question Pro to develop an online survey that Presbyterian Hospital IRB Committee approved. The estimated completion time is less than five minutes. The survey is modeled after previously established surveys by Dr. Rajiv Samant and Dr. Evan Siau [7,8]. The survey asked participants about the following: year of graduation from residency, previous training in RO, board certification, work scope with cancer patients (inpatient or outpatient), self-rated knowledge of RT, and factors that influence their decision whether to refer for RT. It also objectively assessed knowledge of RT by asking participants to select whether RT is “very effective,” “somewhat effective,” or “not effective” at treating common cancer-related symptoms. The correct answer was “very effective” for painful bone metastasis and spinal cord compression; “not effective” for febrile neutropenia, lymphedema, and hypercalcemia; and both “somewhat effective” and “very effective” were accepted as correct for the remaining symptoms (superior vena cava obstruction, brain metastases, dysphagia, and vaginal bleeding) [7]. The survey has a total of 27 questions.

Questionnaire distribution

The survey was distributed anonymously through PHS (Presbyterian Healthcare Services) Microsoft Outlook email groups after obtaining permission from Hospitalist' Medical Director. The email contained link to Question Pro website where the survey is hosted. After the initial survey link was sent, monthly email reminders were sent for three emails. All results were pooled into data analysis.

Statistical analysis

All results were pooled into data analysis. We used descriptive and analytic statistics using Statistical Package for the Social Sciences IBM-SPSS 26 (IBM Corp., Armonk, NY). Mean and standard deviation were used for continuous scale variables, and frequency and percentages for categorical variables. Summarized data was presented as n (%) for all the qualitative questions. The Chi-square (χ2) tests were implemented to test the association between questions at a 5% level of significance.

## Results

An email invitation to complete the survey was sent to 242 potential participants, and 40 responded (17%). 89.7% of respondents (35/39) work primarily as hospitalists, 10.3% (4/39) were primary care physicians, and one did not disclose this information. Approximately more than half of respondents (67.5%) are trained in Internal Medicine (IM), 97.5% are board certified, and only one respondent had prior oncology training. The summary of respondents' demographics is reflected in Table 1.

**Table 1:** Respondents' training and education demographics

Questions	Answers	N	%
Years of graduation from formal training such as residency and/or non-oncology fellowship	1-5 years	18	45.0
	5-10	12	30.0
	more than 10	10	25.0
Are you practicing mostly inpatient?	Yes	35	89.7
	No	4	10.3
Have you referred your cancer patients to radiation oncology before?	Yes	20	50.0
	No	20	50.0
What residency did you graduate from?	Family medicine	13	32.5
	Internal medicine	27	67.5
Have you done additional CME training in general oncology and/or radiation oncology?	Yes	1	2.5
	No	39	97.5
Are you board certified in your specialty?	Yes	39	97.5
	No	1	2.5

**Figure 1:**
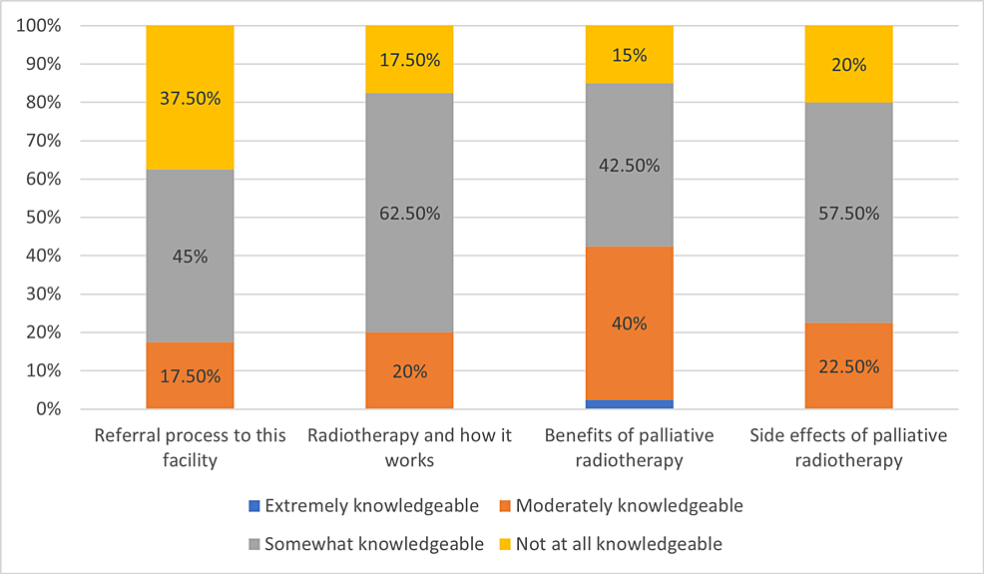
Self rated knowledge

Participants' self-assessed knowledge is reflected in Figure 1. Having a “somewhat knowledgeable” level to “moderately knowledgeable” ranged from 17.5% to 62.5% across the board when it comes to knowledge regarding how radiotherapy works to the referral process. One respondent scored themselves as “extremely knowledgeable” about the benefits of palliative radiotherapy, and the highest percentage of “not at all knowledgeable” (37.5%) was scored in the referral process. 

**Figure 2:**
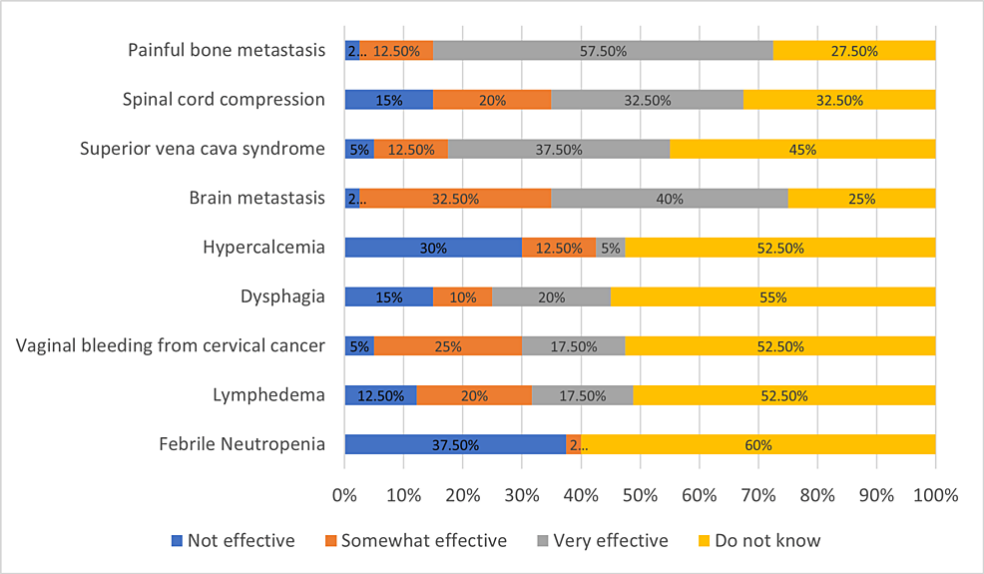
Objective assessment of radiation therapy (RT) efficacy

Another part of the survey objectively assesses the respondents' knowledge with questions about RT's efficacy in common cancer symptoms. For febrile neutropenia, lymphedema, and hypercalcemia, the accepted answer was “not effective,” and only less than 50% of participants answered correctly. For painful bone metastasis, the right answer was “very effective,” and 57.5% answered correctly. The spinal cord compression question also has “very effective” as the right answer, although only 32% answered correctly. The highest “do not know” answer was for lymphedema at 65%, and the lowest was for brain metastasis at 25%. The full results are summarized in Figure 2.

**Figure 3:**
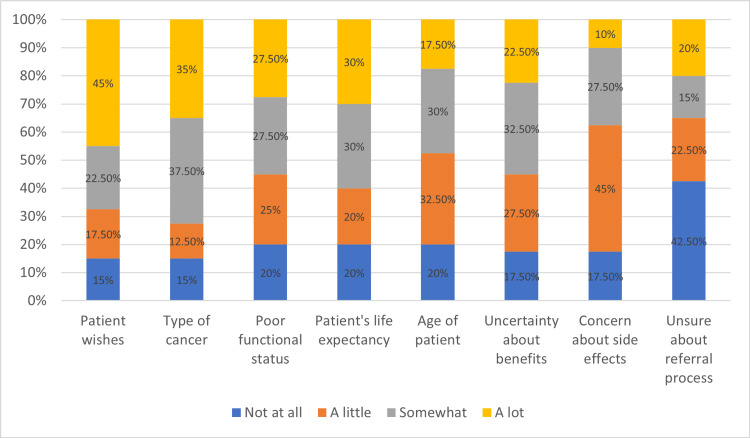
Factors that influence referral

Patient wishes are perceived as the strongest factor influencing referral to RO, and being unsure about the referral process is the least strong factor. Figure 3 summarizes the factors that influence participants' decision to refer.

Recent and older graduates do not have significant differences in their self-rated knowledge of RT, palliative radiotherapy, and the referral process at this facility. Respondents who had referred their patients to RO before rated their knowledge highly in the referral process (p-value < 0.05). No significant difference is found in respondents who trained in IM versus Family Medicine. Additional training in RO and/or medical oncology, as well as board certification in their specialty, does not confer statistical difference in the respondents' self-rated knowledge as well. However, it is worth noting that 97.5% of respondents have not had additional training in oncology despite being board certified in their specialty (Table 2).

**Table 2:** Cross-tabulation for self-rated knowledge

Questions	Categories	Extremely knowledgeable	Moderately knowledgeable	Somewhat knowledgeable	Not at all knowledgeable	P-value
Referral process knowledge						
Years of graduation from formal training	1-5 years	0	2	8	8	0.758
		0.0%	28.6%	44.4%	53.3%	
	5-10	0	2	6	4	
		0.0%	28.6%	33.3%	26.7%	
	more than 10	0	3	4	3	
		0.0%	42.9%	22.2%	20.0%	
Radiotherapy and how it works						
Years of graduation from formal training	1-5 years	0	3	11	4	0.866
		0.0%	37.5%	44.0%	57.1%	
	5-10	0	2	8	2	
		0.0%	25.0%	32.0%	28.6%	
	more than 10	0	3	6	1	
		0.0%	37.5%	24.0%	14.3%	
Benefits of palliative radiotherapy						
Years of graduation from formal training	1-5 years	0	7	8	3	0.774
		0.0%	43.8%	47.1%	50.0%	
	5-10	0	5	5	2	
		0.0%	31.3%	29.4%	33.3%	
	more than 10	1	4	4	1	
		100.0%	25.0%	23.5%	16.7%	
Side effects of palliative radiotherapy						
Years of graduation from formal training	1-5 years	0	3	11	4	0.188
		0.0%	33.3%	47.8%	50.0%	
	5-10	0	1	8	3	
		0.0%	11.1%	34.8%	37.5%	
	more than 10	1	5	4	1	
		100.0%	55.6%	17.4%	12.5%	
Referral process						
Are you practicing mostly inpatient?	Yes	0	6	16	13	0.652
		0.0%	100.0%	88.9%	86.7%	
	No	0	0	2	2	
		0.0%	0.0%	11.1%	13.3%	
Radiotherapy and how it works						
Are you practicing mostly inpatient?	Yes	0	7	21	7	0.614
		0.0%	87.5%	87.5%	100.0%	
	No	0	1	3	0	
		0.0%	12.5%	12.5%	0.0%	
Benefits of palliative radiotherapy						
Are you practicing mostly inpatient?	Yes	1	15	13	6	0.506
		100.0%	93.8%	81.3%	100.0%	
	No	0	1	3	0	
		0.0%	6.3%	18.8%	0.0%	
Side effects of palliative radiotherapy						
Are you practicing mostly inpatient?	Yes	0	8	19	8	0.550
		0.0%	88.9%	86.4%	100.0%	
	No	0	1	3	0	
		0.0%	11.1%	13.6%	0.0%	
Radiation oncology referral process at this facility						
Have you referred your cancer patients to radiation oncology before?	Yes	0	6	10	4	0.029
		0.0%	85.7%	55.6%	26.7%	
	No	0	1	8	11	
		0.0%	14.3%	44.4%	73.3%	
Radiotherapy and how it works						
Have you referred your cancer patients to radiation oncology before?	Yes	0	5	13	2	0.401
		0.0%	62.5%	52.0%	28.6%	
	No	0	3	12	5	
		0.0%	37.5%	48.0%	71.4%	
Benefits of palliative radiotherapy						
Have you referred your cancer patients to radiation oncology before?	Yes	1	10	8	1	0.193
		100.0%	62.5%	47.1%	16.7%	
	No	0	6	9	5	
		0.0%	37.5%	52.9%	83.3%	
side effects of palliative radiotherapy						
Have you referred your cancer patients to radiation oncology before?	Yes	0	6	13	1	0.053
		0.0%	66.7%	56.5%	12.5%	
	No	0	3	10	7	
		0.0%	33.3%	43.5%	87.5%	
radiation oncology referral process at this facility						
What residency did you graduate from?	Family medicine	0	2	6	5	0.971
		0.0%	28.6%	33.3%	33.3%	
	Internal medicine	0	5	12	10	
		0.0%	71.4%	66.7%	66.7%	
Radiotherapy and how it works						
What residency did you graduate from?	Family medicine	0	1	9	3	0.379
		0.0%	12.5%	36.0%	42.9%	
	Internal medicine	0	7	16	4	
		0.0%	87.5%	64.0%	57.1%	
benefits of palliative radiotherapy						
What residency did you graduate from?	Family medicine	0	4	6	3	0.617
		0.0%	25.0%	35.3%	50.0%	
	Internal medicine	1	12	11	3	
		100.0%	75.0%	64.7%	50.0%	
side effects of palliative radiotherapy						
What residency did you graduate from?	Family medicine	0	2	7	4	0.450
		0.0%	22.2%	30.4%	50.0%	
	Internal medicine	0	7	16	4	
		0.0%	77.8%	69.6%	50.0%	
Radiation oncology referral process at this facility						
Have you done additional CME training in general oncology and/or radiation oncology?	Yes	0	0	0	1	0.425
		0.0%	0.0%	0.0%	6.7%	
	No	0	7	18	14	
		0.0%	100.0%	100.0%	93.3%	
Radiotherapy and how it works						
Have you done additional CME training in general oncology and/or radiation oncology?	Yes	0	1	0	0	0.129
		0.0%	12.5%	0.0%	0.0%	
	No	0	7	25	7	
		0.0%	87.5%	100.0%	100.0%	
Benefits of palliative radiotherapy						
Have you done additional CME training in general oncology and/or radiation oncology?	Yes	0	1	0	0	0.673
		0.0%	6.3%	0.0%	0.0%	
	No	1	15	17	6	
		100.0%	93.8%	100.0%	100.0%	
Side effects of palliative radiotherapy						
Have you done additional CME training in general oncology and/or radiation oncology?	Yes	0	1	0	0	0.171
		0.0%	11.1%	0.0%	0.0%	
	No	0	8	23	8	
		0.0%	88.9%	100.0%	100.0%	
Radiation oncology referral process at this facility						
Are you board certified in your specialty?	Yes	0	7	18	14	0.425
		0.0%	100.0%	100.0%	93.3%	
	No	0	0	0	1	
		0.0%	0.0%	0.0%	6.7%	
Radiotherapy and how it works						
Are you board certified in your specialty?	Yes	0	8	25	6	0.089
		0.0%	100.0%	100.0%	85.7%	
	No	0	0	0	1	
		0.0%	0.0%	0.0%	14.3%	
Benefits of palliative radiotherapy						
Are you board certified in your specialty?	Yes	1	16	17	5	0.121
		100.0%	100.0%	100.0%	83.3%	
	No	0	0	0	1	
		0.0%	0.0%	0.0%	16.7%	
Side effects of palliative radiotherapy						
Are you board certified in your specialty?	Yes	0	9	23	7	0.129
		0.0%	100.0%	100.0%	87.5%	
	No	0	0	0	1	
		0.0%	0.0%	0.0%	12.5%	

37.5% of participants answered correctly for febrile neutropenia, 12.5% for lymphedema and 30% responded correctly for hypercalcemia. More than half of respondents answered for painful bone metastasis correctly, and 32.5% responded correctly for spinal cord compression. For the remaining symptoms, the percentage of correct answers ranged from 30% (dysphagia) to 72.5% (brain metastasis).

## Discussion

Approximately 25% of patients living with cancer accounted for ED visits due to complications from cancer [13]. Those patients who ended up being hospitalized may have increased morbidity and mortality [15]. The medical community well accepts the hospitalists' role in providing inpatient care to cancer patients [16,17]. A study conducted at Memorial Sloan Kettering Cancer Center GI oncology unit, which compared a hospitalist team versus an oncologist-led team, showed no significant difference in length of stay and readmission rates in both teams [18]. A different study at the University of Texas MD Anderson Cancer Center demonstrated similar results, further affirming the evidence that hospitalists play a significant role in inpatient cancer care, improve outcomes, and even can be cost saving [19]. RO is an integral part of cancer care, and as hospitalists coordinating care is the keystone of prompt management. Prompt referral to RO before discharge is vital since care delay could potentially harm patients. Despite this, little to no studies have been done to assess the knowledge of hospitalists regarding RO, especially in a nonacademic private healthcare setting. This study showed that 17.5% to 62.5% of respondents scored themselves as “somewhat knowledgeable” level to “moderately knowledgeable” when it comes to knowledge regarding how radiotherapy works in the referral process. Despite having referred their patients to RO before, most hospitalists do not think they have sufficient knowledge in this field, consistent with prior studies examining RO knowledge among generalists [20,21]. The highest percentage of “not at all knowledgeable” is shown in the referral process of this facility, and there is a significant difference (p<0.05) between self-perceived knowledge of the RO referral process and if respondents have referred their patients before to RO. Lack of knowledge could be a barrier to the referral process, as shown in a previous study of oncologists and hospice/palliative care physicians that showed nearly 70% of medical oncologists and 80% of hospice/palliative care physicians endorsed “lack of training” as a barrier to the referral process [22].

RO training has been deemed insufficient in physicians both at attendings and trainee levels [7-9]. A study examining the RO curriculum in Europe showed that the hours dedicated to RO teaching are significantly less than in medical oncology [23]. Lack of exposure from the trainee level could be the main reason for self-perceived lack of knowledge at the attending level, especially if there is no additional prior training in Oncology and lack of exposure to cancer patients. Being recent versus older graduate, having a type of residency, and having board certification does not seem to have a significant difference in the level of self-perceived knowledge which is a contrast to Dr. Siau et al.'s survey that showed a higher score of objective assessment in IM residents [7].

On objective assessment, approximately more than half of respondents answered correctly for painful bone metastasis, and an even higher percentage (72.5%) answered correctly for brain metastasis, like answers from previous surveys of GPs, community hospital residents, and hospice physicians [7,22,24,25]. However, only less than 50% answered correctly for the remaining symptoms. Painful bone metastasis and brain metastasis are common complications of cancer patients [26-28], which could explain the higher score of correct answers; however, spinal cord compression from metastasis is not significantly less common than brain metastasis [29], yet the score is lower. An argument could be made that the respondents do not see these cases in their daily rounds. As a nonacademic healthcare system, there is less exposure to academic requirements such as publication, journal club, and grand rounds, which leads to less training exposure.

Patients' wishes and type of cancer were cited as the most important influencing factors for referral to RT (85%), followed by uncertainty about benefits and side effects in no particular order (82.5%). Age, poor functional status, and life expectancy all have the same rate of 80%, and being unsure about the referral process is the lowest at 57.5%. It is reassuring to see that the respondents do not think being unsure about the referral process is an important factor in establishing a referral, and honoring patients' wishes is extremely important, which is consistent with previously established studies [7,8,24]. It is also worth noting that the referral process was scored as the least knowledgeable item in the self-rated knowledge section. Despite this, the respondents think this is not important in establishing referrals.

This study has some limitations. The small sample size means the results could not be applied to the general population. This study was also conducted in a healthcare system that does not cater specifically to cancer patients; therefore, the cases will be limited. This study's respondents also only consisted of adult physicians; therefore, the pediatric population is excluded. The amount of respondents who practiced primarily as PCPs were also quite low therefore it is not able to reflect the population of PCP in general. Despite its limitations, this study is one of the few studies that assessed RO knowledge among generalists and, as far as the authors are aware, the only study that specifically assessed hospitalists' knowledge. This study is also unique in that it is conducted in a large healthcare system that serves a broad range of patients with extensive illnesses despite not having academic affiliations. Further studies could be explicitly replicated for hospitalists in both academic and nonacademic settings, and the results could further support training and education in RO for hospitalists.

## Conclusions

Many hospitalists who took this survey do not have sufficient knowledge of RO and the referral process despite having cared for cancer patients before. There is sufficient evidence that generalist physicians do not have enough training in RO despite the clearly established role of RT in comprehensive cancer care. A prompt referral process to RO before discharging cancer patients is important for the patients. Increasing educational efforts for hospitalists in RO would certainly be beneficial.
